# Construction of influencing factors and nomogram prediction model for post-herpetic neuralgia based on T cell function and inflammatory factors

**DOI:** 10.3389/fmed.2025.1619157

**Published:** 2025-06-18

**Authors:** Lin Liu, Shunqiang Chen

**Affiliations:** ^1^Department of Painology, Henan Provincial People’s Hospital, Zhengzhou, China; ^2^Department of Radiology, Henan Provincial People’s Hospital, Zhengzhou, China

**Keywords:** herpes zoster, post herpetic neuralgia, T cell function, inflammatory factors, prediction models

## Abstract

**Objective:**

To explore the feasibility and clinical value of establishing a prediction model of post-herpetic neuralgia (PHN) based on T cell functional indicators (CD4+/CD8+ ratio, Treg cell ratio) and inflammatory factors (IL-6, TNF-*α*, and IL-10).

**Methods:**

A total of 260 patients with herpes zoster who were admitted to our hospital from June 2022 to November 2024 were included in the study. The 7:3 score was used as the training set (*n* = 182) and verification set (*n* = 78). The clinical data were collected and the peripheral blood T cell subsets and inflammatory factor levels were detected. Risk factors were screened by univariate and multivariate Logistic regression, and a nomogram model was constructed for efficacy evaluation and verification.

**Results:**

The incidence of PHN in the training set was 29.67%(54/182) and the verification set was 30.77%(24/78). Multivariate regression analysis showed that age, CD4+/CD8+ ratio, Treg cell ratio, IL-6, TNF-*α*, and IL-10 were the independent risk factors (*p* < 0.05). The C-index values for the nomogram models in the training and validation sets were 0.804 and 0.789, respectively, the AUC values were 0.802 (95% CI: 0.722–0.882) and 0.790 (95% CI: 0.642–0.938), and the sensitivity and specificity values were 0.634, 0.875, and 0.462 and 0.875, respectively. The calibration curve showed good agreement between the predicted and actual values with mean absolute errors of 0.164 and 0.146, respectively, which was good by the Hosmer-Lemeshow test.

**Conclusion:**

The nomogram model based on T cell function and inflammatory factors can effectively predict the risk of PHN and provide the basis for early clinical intervention.

## Introduction

Herpes zoster (Hz) is an acute infectious skin disease caused by the reactivation of varicella-zoster virus (VZV), which is characterized by unilateral clusters of blisters with severe pain (1). Post-herpetic neuralgia (PHN), as the most common and severe chronic complication of HZ, refers to the pain that lasts for more than 3 months after the rash resolves, often presenting as burning-like, electric shock-like or tear-like pain, which has seriously affected the quality of life of patients (2). Epidemiological studies have shown that the incidence of PHN increases significantly with age, and the incidence in patients over the age of 60 can reach 30–50%. In addition, the longer the duration of pain, the more difficult the treatment is. It has become an important public health problem (3). At present, the risk factors of PHN have been widely concerned, including the elderly, women, rash severity, pain intensity in the acute phase, and immunosuppressive state. However, the pathogenesis has not yet been fully clarified. There is increasing evidence that immune system imbalance plays a key role in the development of PHN (4). As the core component of anti-VZV immunity, T cells’ dysfunction may lead to delayed virus clearance and persistent nerve damage. The CD4+/CD8+ ratio reflects the dynamic balance between helper T cells and cytotoxic T cells, and a decrease in the ratio may indicate an insufficient antiviral immune response. An increased proportion of regulatory T cells (Treg cells) may exacerbate persistent viral infection by inhibiting the immune response. In addition, inflammatory factors such as interleukin-6 (IL-6) and tumor necrosis factor-*α* (TNF-α) are involved in the neuroinflammatory response, while IL-10, as an anti-inflammatory factor, its abnormal elevation may interfere with the immune repair process. However, the existing studies mostly focus on the association between a single immune indicator and PHN, lack the integrated analysis of T cell function and inflammatory factors, and the prediction model based on immune indicators has not been reported yet.

## Materials and methods

### Materials

A total of 260 patients with herpes zoster were selected from June 2022 to November 2024 in our hospital. Inclusion criteria: (1) Patients met the diagnostic criteria stipulated in Guideline for Diagnosis and Treatment of Herpes Zoster (5); (2) Age ≥18 years old; (3) First occurrence of disease and no antiviral treatment. Exclusion criteria: (1) concomitant malignant tumor; (2) Autoimmune diseases; (3) Severe hepatic and renal insufficiency; (4) Pregnant and lactating women. The training set (*n* = 182) and verification set (*n* = 78) were divided according to 7:3 points by hierarchical random method. (5) History of HIV infection or other acquired immunodeficiency diseases; (6) Current use of immunosuppressive therapy (e.g., corticosteroids, chemotherapy, biologics); (7) Hematologic malignancies (e.g., leukemia, lymphoma). This study was approved by the Ethics Committee of Henan Provincial People’s Hospital.

### Data collection

The clinical data such as age, gender, basic diseases (diabetes, hypertension, etc.), and rash site were recorded. Venous blood was collected at the time of admission. The proportions of peripheral blood CD4+, CD8+ T cells and Treg cells (defined as CD4+ CD25+ FoxP3+ cells as a percentage of total CD4+ T cells) were detected by flow cytometry, and the levels of IL-6, TNF-*α*, and IL-10 were detected by enzyme-linked immunosorbent assay (ELISA). Flow cytometry gating strategies were as follows: peripheral blood mononuclear cells were first gated by forward/side scatter, followed by identification of CD3+ T cells. CD4+ and CD8+ T cell subsets were then defined within the CD3+ population. Treg cells were further identified as CD4+ CD25+ FoxP3+ cells within the CD4+ T cell subset using fluorescence-conjugated antibodies (CD3-FITC, CD4-PE, CD25-APC, FoxP3-PE/Cy7; BD Biosciences, United States). The duration of symptoms (defined as the number of days from the onset of rash to hospital admission) was recorded for all patients. Detailed records of comorbidities (e.g., diabetes, hypertension) and prior immunosuppressive conditions were collected to ensure adherence to exclusion criteria.

### Diagnostic criteria

Diagnostic criteria for PHN: Pain persisted for ≥3 months after the rash resolved, and Visual Analog Scale (VAS) score ≥ 4 points.

### Statistical methods

SPSS25.0 and R4.0.3 software were used for data analysis. Measurement data were expressed as mean standard deviation. Independent sample t test was used for comparison between two groups, and analysis of variance was used for comparison among multiple groups. The count data were expressed as number of cases and percentage (*n*,%), and the comparison between groups was performed by *χ* test. Logistic regression analysis was used to screen the independent risk factors affecting the curative effect of treatment. Application of r software to construct nomograph prediction model based on independent risk factors. The receiver operating characteristic (ROC) curve was used to evaluate the prediction performance of the model, and the Area Under Curve (AUC) and 95% Confidence Interval (CI) were calculated. A calibration curve was used to evaluate the consistency of the model predictions with the actual observations. *p* < 0.05 was considered as the difference with statistical significance.

## Results

### Comparison of patient baseline basic information between training and verification sets

There were no significant differences in clinical data such as age, gender, underlying disease, and rash site between the training set (*n* = 182) and the verification set (*n* = 78) (*p* > 0.05), indicating that they were comparable (Table 1).

**Table 1:** Comparison of patient baseline profile for training and validation sets.

Index		Training set (*n* = 182)	Validation set (*n* = 78)	Statistical values	*p*
Age		58.32 ± 11.58	56.88 ± 10.28	0.949	0.343
Gender	Man	95 (52.20)	42 (53.85)	0.059	0.807
	Woman	87 (47.80)	36 (46.15)		
History of diabetes	Yes	50 (27.47)	19 (24.36)	0.271	0.603
	No	132 (72.53)	59 (75.64)		
History of hypertension	Yes	71 (39.01)	32 (41.03)	0.093	0.761
	No	111 (60.99)	46 (58.97)		
Rash site	Head	47(25.82)	22 (28.21)	0.159	0.691
	Face	135(74.18)	56 (71.79)		
Antiviral treatment delayed (Days)		4.12 ± 2.32	4.28 ± 2.01	0.529	0.597
CD4+/CD8+ ratio		1.18 ± 0.40	1.23 ± 0.68	0.739	0.461
Treg cell proportion		4.85 ± 1.51	4.63 ± 1.23	1.135	0.257
IL-6 (pg/mL)		20.02 ± 5.05	21.23 ± 4.86	1.791	0.075
TNF-α (pg/mL)		37.41 ± 7.21	35.68 ± 6.89	1.796	0.073
IL-10 (pg/mL)		16.88 ± 5.23	15.43 ± 6.82	1.863	0.064
Lymphocyte count (× 10/L)		1.37 ± 0.61	1.32 ± 0.89	0.524	0.601
Neutrophil count (× 10/L)		5.13 ± 2.01	5.23 ± 2.31	0.351	0.726
Platelet count (× 10/L)		300.21 ± 82.82	302.65 ± 96.52	0.215	0.831
Hemoglobin (g/L)		129.61 ± 16.22	130.25 ± 20.25	0.269	0.787
Serum vitamin D (ng/mL)		20.51 ± 8.12	22.31 ± 7.62	1.668	0.097
Symptom Duration (Days)		4.12 ± 2.32	4.28 ± 2.01	0.529	0.597

CD4+/CD8+ ratio and Treg cell proportion were measured in peripheral blood. All enrolled patients were screened for HIV infection, immunosuppressive therapy, and hematologic malignancies as per exclusion criteria; no such cases were included in the final cohort. Take home message: The training set (*n* = 182) and validation set (*n* = 78) showed no significant differences in baseline characteristics such as age, gender, comorbidities, rash location, or immune/inflammatory markers (all *p* > 0.05), confirming the comparability of the two cohorts for subsequent model development and validation.

### Univariate analysis of risk factors for post-herpetic neuralgia in patients from the training set

In the training set, there were 54 cases (29.67%) with PHN and 128 cases (70.33%) without PHN. Univariate analysis showed that there were significant differences in age, CD4+/CD8+ ratio, Treg cell ratio, IL-6, TNF-*α*, and IL-10 between the PHN group and the non-PHN group (*p* < 0.05), as shown in Table 2.

**Table 2:** Univariate analysis of risk factors for postherpetic neuralgia in patients of training set.

Index	PHN occurring (*n* = 54)		PHN not occurring (*n* = 128)	Statistical values	*p*
Age		62.31 ± 10.52	55.42 ± 12.71	3.507	0.001
Gender	Man	23 (42.59)	72 (56.25)	2.839	0.092
	Woman	31 (57.41)	56 (43.75)		
History of diabetes	Yes	20 (37.04)	30 (23.44)	3.525	0.061
	No	34 (62.96)	98 (76.56)		
History of hypertension	Yes	23 (42.59)	48 (37.50)	0.414	0.519
	No	31 (57.41)	80 (62.50)		
Rash site	Head	19 (35.19)	28 (21.88)	3.512	0.061
	Face	35 (64.81)	100 (78.13)		
Antiviral treatment delayed (Days)		4.32 ± 2.12	3.88 ± 1.56	1.555	0.122
CD4+/CD8+ ratio		1.08 ± 0.43	1.28 ± 0.44	2.819	0.005
Treg cell proportion		4.55 ± 1.21	5.23 ± 1.52	2.919	0.004
IL-6 (pg/mL)		21.02 ± 5.85	18.96 ± 3.52	2.926	0.004
TNF-α (pg/mL)		38.42 ± 6.21	35.21 ± 8.76	2.444	0.016
IL-10 (pg/mL)		18.18 ± 5.63	15.76 ± 4.82	2.941	0.004
Lymphocyte count (× 10/L)		1.27 ± 0.51	1.52 ± 0.89	1.933	0.055
Neutrophil count (× 10/L)		5.53 ± 2.11	4.96 ± 1.89	1.795	0.074
Platelet count (× 10/L)		310.23 ± 85.12	290.23 ± 70.69	1.638	0.103
Hemoglobin (g/L)		128.63 ± 15.12	132.16 ± 18.21	1.253	0.212
Serum vitamin D (ng/mL)		19.54 ± 7.12	21.81 ± 8.01	1.803	0.073
Symptom duration (Days)		4.32 ± 2.23	4.02 ± 2.25	0.991	0.322

Take home message: Univariate analysis identified six variables (age, CD4+/CD8+ ratio, Treg cell proportion, IL-6, TNF-*α*, and IL-10) as significantly associated with PHN occurrence (*p* < 0.05), providing preliminary evidence for their inclusion in multivariate modeling.

### Multi-factor logistic regression analysis of patients with post-herpetic neuralgia

PHN was used as the dependent variable (0 = none, 1 = occurrence), the factor *p* < 0.05 in the single factor analysis was used as the covariate, and the variable assignments were shown in Table 3. The results of multi-factor Logistic regression analysis showed that age, CD4+/CD8+ ratio, Treg cell ratio, IL-6, TNF-*α*, and IL-10 were the independent risk factors for postherpetic neuralgia (*p* < 0.05). In the regression model, the tolerance of each variable was > 0.1, VIF was <10, condition index was <30, and the proportion of variances of multiple covariates was > 50% without the same feature value. Hence, there was no collinearity of each covariate, as shown in Table 4.

**Table 3:** Variable assignment method.

Variable	Meaning	Evaluation
X1	Age	Continuous variable
X2	CD4+/CD8+ ratio	Continuous variable
X3	Treg cell proportion	Continuous variable
X4	IL-6	Continuous variable
X5	TNF-α	Continuous variable
X6	IL-10	Continuous variable
Y	PHN	Occurrence = 1, none = 0

**Table 4:** Results of multivariate logistic regression analysis.

Project	B	SE	Wald	*p*	OR	95% confidence interval
Age	0.058	0.017	11.495	0.001	1.060	1.025–1.096
CD4+/CD8+ ratio	−0.988	0.434	5.187	0.023	0.372	0.159–0.871
Treg cell proportion	−0.379	0.141	7.199	0.007	0.685	0.519–0.903
IL-6	0.132	0.042	9.843	0.002	1.141	1.051–1.239
TNF-α	0.051	0.025	4.139	0.042	1.052	1.002–1.105
IL-10	0.104	0.039	7.142	0.008	1.110	1.028–1.105

Take home message: Multivariate logistic regression confirmed age (OR = 1.060), CD4+/CD8+ ratio (OR = 0.372), Treg cell proportion (OR = 0.685), IL-6 (OR = 1.141), TNF-α (OR = 1.052), and IL-10 (OR = 1.110) as independent risk factors for PHN (all P < 0.05), with no evidence of multicollinearity among variables (tolerance > 0.1, VIF < 10).

### Construction of nomogram prediction model

**Figure 1:**
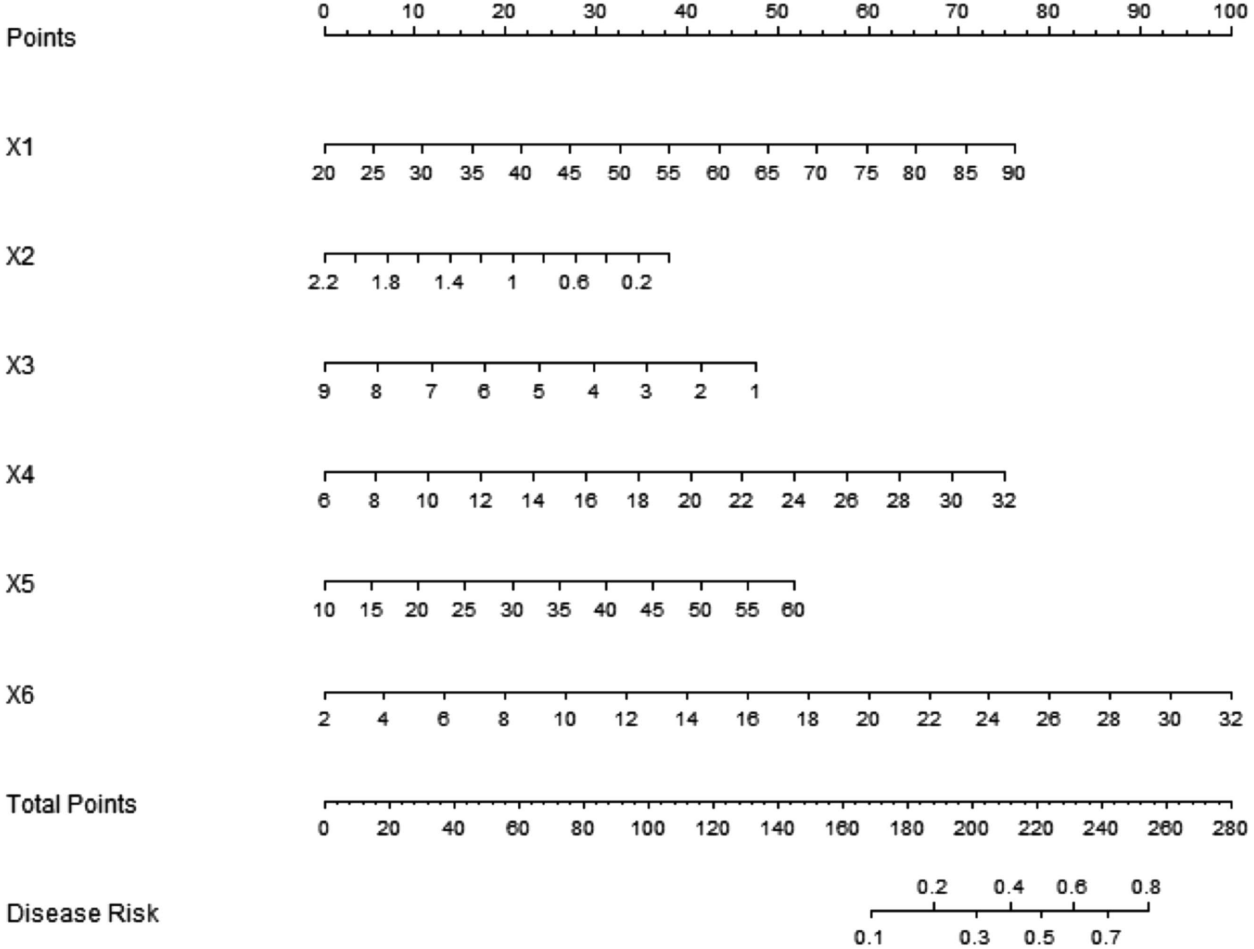
Construction of an nomogram prediction model for post-herpetic neuralgia based on T cell function and inflammatory factors. x1, age; X2, CD4+/CD8+ ratio; X3, proportion of Treg cells; x4, IL-6; x5: TNF-*α*; x6: IL-10. Take home message: The nomogram model integrated six independent risk factors into a visual scoring tool, enabling individualized prediction of PHN risk by summing weighted contributions from age, T cell subsets, and inflammatory cytokines.

Based on the independent risk factors determined by multivariate Logistic regression analysis, an nomogram prediction model for post-herpetic neuralgia was constructed based on T cell function and inflammatory factors. Each independent risk factor in the model was scored, and the total score of predicted IMBD was calculated, which was expressed as the prediction probability, as shown in Figure 1.

### Evaluation and validation of nomogram prediction model

**Figure 2:**
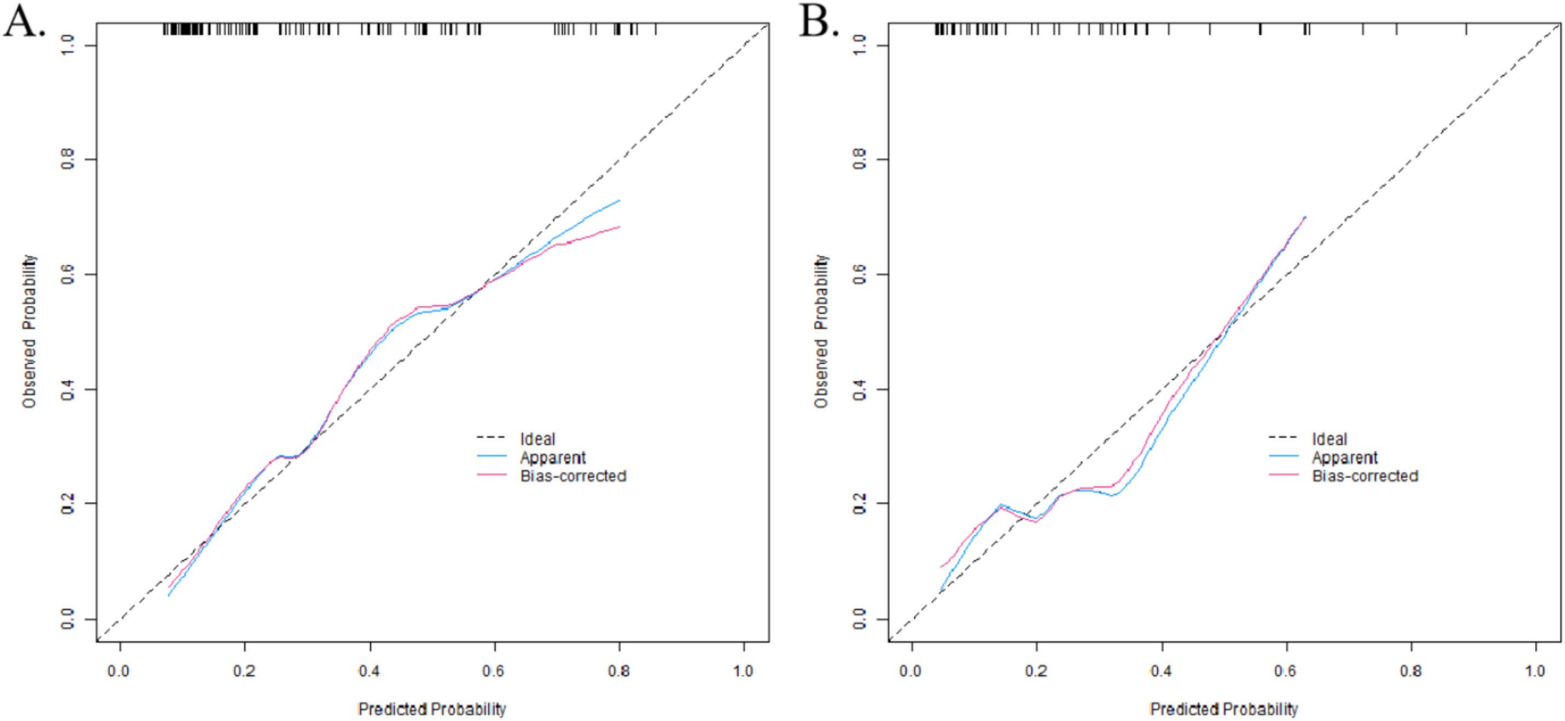
Calibration curve (**A** training set, **B** verification set).

**Figure 3:**
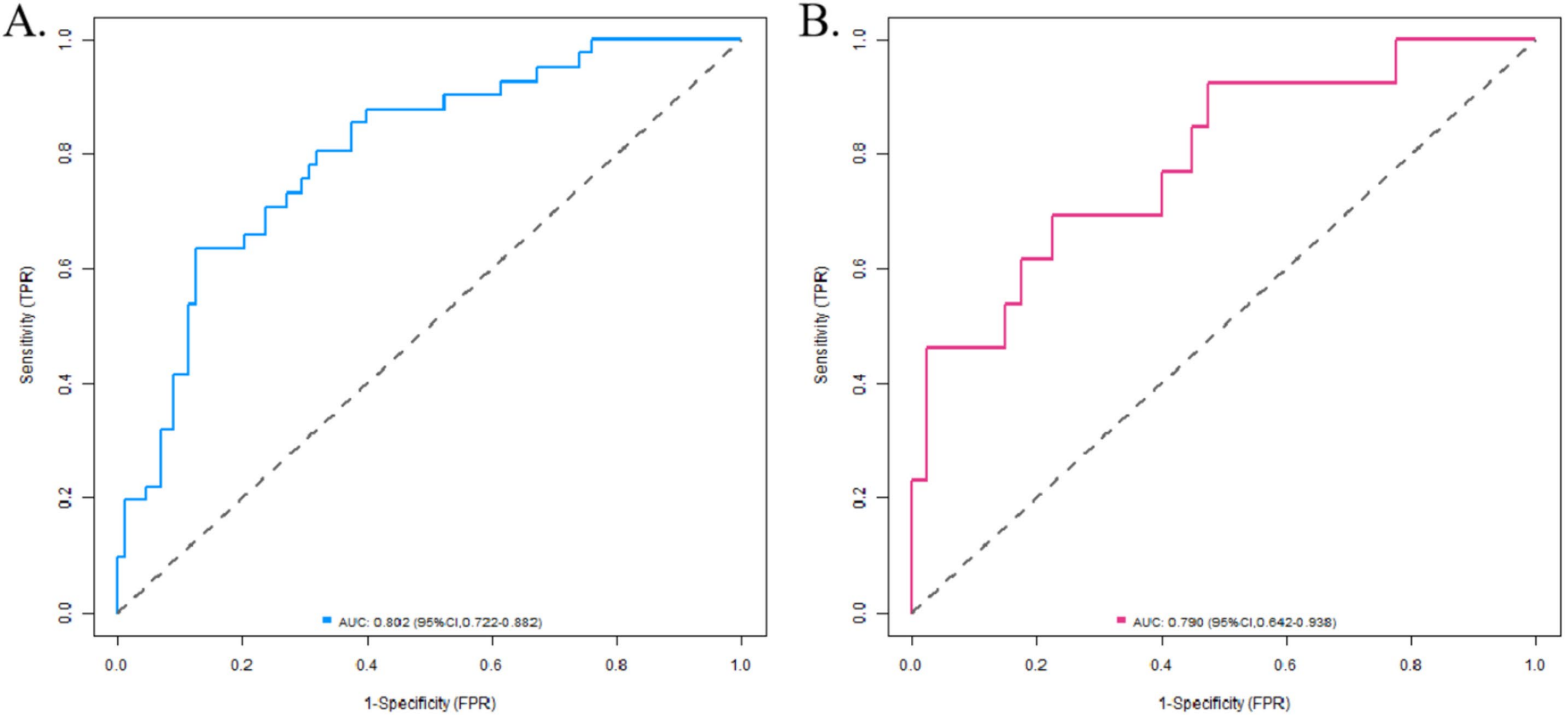
ROC curve (**A** training set, **B** verification set). Take home message: The nomogram demonstrated robust predictive performance in both training (C-index = 0.804, AUC = 0.802) and validation sets (C-index = 0.789, AUC = 0.790), with calibration curves and Hosmer-Lemeshow tests (*p* > 0.05) indicating excellent agreement between predicted and observed outcomes.

In the training set, the C-index index of the nomogram prediction model was 0.804, the average absolute error of the predicted value and the actual value in the calibration curve was 0.164, and the *p* = 0.529 by Hosmer-Lemeshow test indicated that the model fitted well. The ROC curve showed the model predicted AUC to be 0.802 (95% CI: 0.722–0.882), sensitivity to be 0.634, and specificity to be 0.875. In the validation set, the C-index was 0.789, the mean absolute error was 0.146, the Hosmer-Lemeshow test *p* = 0.459, the AUC was 0.790 (95% CI: 0.642–0.938), the sensitivity was 0.462, and the specificity was 0.875. The calibration curve and ROC curve are shown in Figures 2, 3 respectively.

### Decision curve analysis

**Figure 4:**
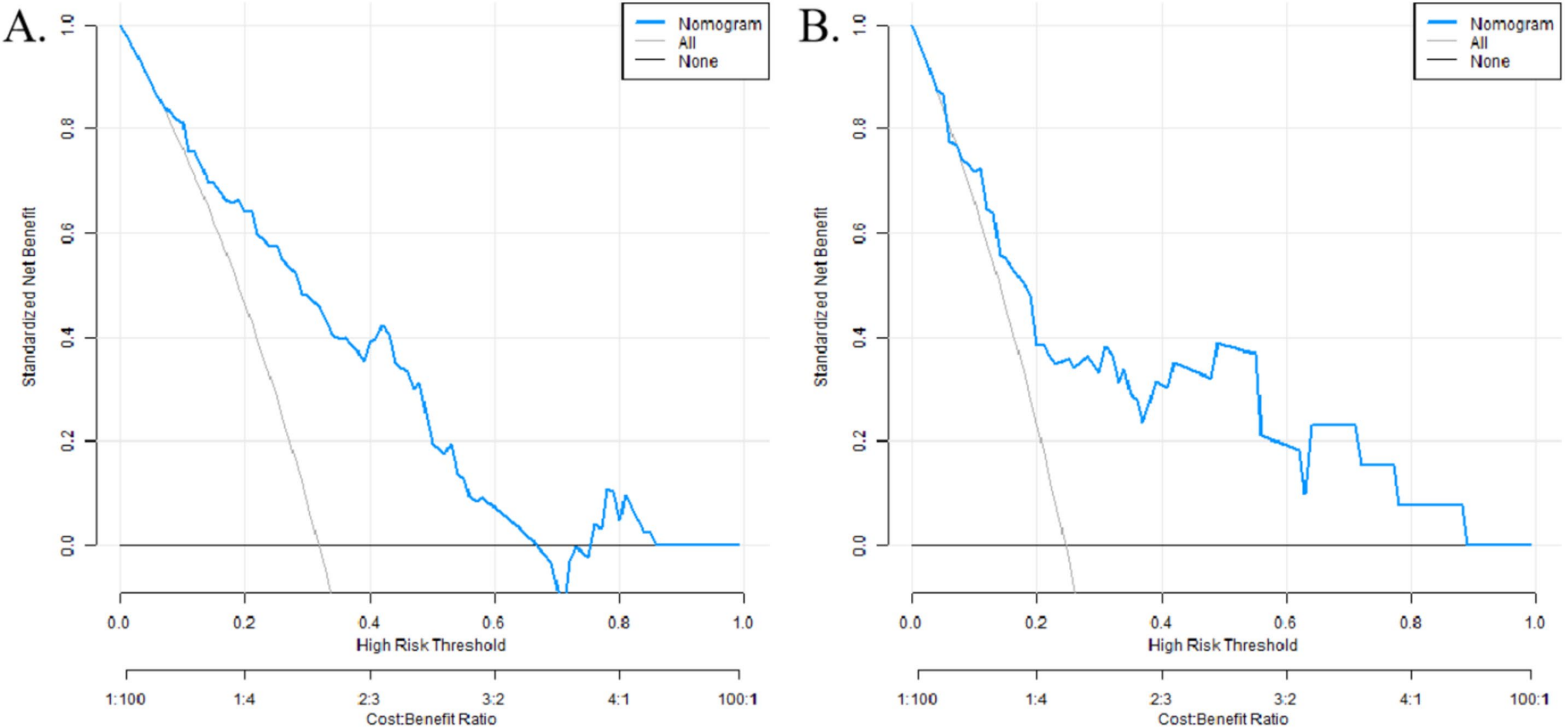
Decision curve (**A** training set, **B** verification set). Take home message: Decision curve analysis confirmed the clinical utility of the nomogram, showing a net benefit across threshold probabilities of 0.10–0.90, thereby supporting its application for risk stratification and early intervention in PHN management.

The analysis of decision curves showed that when the threshold probability was within the range of 0.10–0.90, the nomogram model constructed in this study had the best net benefit for predicting the decision-making for postherpetic neuralgia, as shown in Figure 4.

## Discussion

PHN remains a major challenge in elderly populations, with age being the strongest immutable risk factor [OR = 1.060 per year; (Table 4)]. Our findings further reveal that age synergizes with immune dysfunction (e.g., Treg/IL-10 imbalance) to amplify PHN risk, providing a mechanistic basis for targeted interventions. There is increasing evidence that immune system imbalance plays a key role in the development of PHN, in particular, T cell dysfunction and inflammatory factor imbalance may lead to delayed virus clearance and persistent nerve damage (6). However, the existing studies mostly focus on the association of single immune index with PHN, and lack the integrated analysis of T cell function (such as CD4+/CD8+ ratio, and regulatory T cell Treg ratio) and inflammatory factors (such as IL-6, TNF-*α*, and IL-10). Moreover, prediction models based on immune indexes have not been reported yet. Under this background, the purpose of this study was to construct an nomogram prediction model of PHN by integrating T cell function indicators and inflammatory factors, so as to provide a new tool for early clinical recognition of high-risk patients. The selection of these two indicators was based on the following: T cells are the core components of anti-varicella zoster virus (VZV) immunity, peripheral blood CD4+/CD8+ ratio reflects the dynamic balance between helper T cells and cytotoxic T cells, and a decrease in the ratio may indicate an insufficient anti-viral immune response; A higher proportion of Treg cells may exacerbate persistent viral infection by inhibiting the immune response. As for the inflammatory factors, IL-6 and TNF-*α* are involved in the neuroinflammatory reaction to promote nerve injury, while IL-10, as an anti-inflammatory factor, its abnormally high level may interfere with the immune repair process (7). Through multi-factor analysis and model building, we hope to reveal the synergistic effect of these indicators and fill the gap in the integration and prediction model of immunologic mechanism in the existing research.

We further analyzed the potential confounding effect of symptom duration on inflammatory markers. The comparable symptom duration between PHN and non-PHN groups (Table 1, *p* > 0.05) suggests that the observed differences in IL-6, TNF-*α*, and IL-10 levels are more likely driven by intrinsic immune dysregulation rather than temporal variations in disease progression. This strengthens the hypothesis that T cell dysfunction and cytokine imbalance are central to PHN pathogenesis. Our exclusion of patients with HIV infection, immunosuppressive therapy, or hematologic malignancies aimed to minimize confounding effects from extrinsic immune dysfunction. This design allowed us to focus on age-related immune senescence and intrinsic inflammatory dysregulation as primary contributors to PHN pathogenesis. Prior studies have shown that exogenous immunosuppression (e.g., HIV) drastically alters T cell subsets and cytokine profiles, which could overshadow the subtle immune imbalances observed in immunocompetent PHN patients. By controlling for these factors, our model highlights the unique interplay between physiological immune aging and neuroinflammation in PHN development.

Our multivariate analysis (Table 4) highlighted CD4+/CD8+ ratio and Treg cell proportion as critical immunological predictors of PHN. A lower CD4+/CD8+ ratio (*p* = 0.023, OR = 0.372) and elevated Treg cells (*p* = 0.007, OR = 0.685) synergistically impair antiviral immunity, aligning with prior evidence of T cell dysfunction in chronic neuralgia (8–10). A lower ratio (i.e., a decrease in CD4+ or an increase in CD8+ might result in an insufficient antiviral immune response and delayed viral clearance), which in turn triggers sustained nerve damage. In this study, the CD4+/CD8+ ratio in patients with PHN was significantly lower than that in patients without PHN (1.08vs1.28, *p* = 0.005). Multifactor analysis showed that for every unit that the ratio was decreased, the risk of PHN increased by 62.8%(OR = 0.372), suggesting that the ratio was an important immunological marker for predicting PHN. Regulatory T cells (Treg) maintain immune homeostasis by inhibiting T cell activation, but their overactivation may lead to viral clearance disorders (11). In this study, the proportion of Treg cells in PHN patients was significantly higher than that in the control group (4.55%vs5.23%, *p* = 0.004), and for each 1% increase in proportion, the risk of PHN was reduced by 31.5%(OR = 0.685). This seemingly contradictory result actually reflects the dual roles of Treg cells in chronic inflammation. On the one hand, Treg cells inhibit excessive immune response and reduce tissue damage in the acute phase by secreting anti-inflammatory factors such as IL-10; On the other hand, persistent high expression of Treg cells may inhibit the antiviral immune response, resulting in the long-term incubation of the virus in the ganglion and promotion of the formation of chronic neuralgia. Clinical studies have shown that abnormal recruitment of Treg cells after VZV infection is associated with a reduced virus-specific T-cell response, which provides a mechanism for this study (12). The core of T cell dysfunction lies in the imbalance of immune response (13). Immune aging is common in elderly patients, which is characterized by CD4+ T cell dysfunction and increased proportion of Treg cells, which may explain the synergistic effect of age and T cell indicators. In this study, the risk of PHN increased by 6%(OR = 1.060) for every 1 year old, while the older, the lower CD4+/CD8+ ratio and the higher proportion of Treg cells, which together aggravate the viral clearance disorder and the sustainability of nerve damage (14).

Inflammatory factors play a key role in neuroinflammation and tissue damage in PHN (15). IL-6 and TNF-*α*, as proinflammatory factors, can induce glial cell activation, nerve fiber demyelination and pain sensitization by activating NF-κB signaling pathway (16). This study showed that the levels of IL-6 and TNF-*α* in PHN patients were significantly higher than those in the control group (IL-6: 21.02 vs. 18.96 pg./mL, *p* = 0.004; TNF-α: 38.42 vs. 35.21 pg./mL, *p* = 0.016), multivariate analysis showed that for each 1 pg./mL increase in IL-6, the risk of PHN increased by 14.1%(OR = 1.141), and for each 1 pg./mL increase in TNF-α, the risk increased by 5.2%(OR = 1.052). This result is consistent with previous studies: IL-6 promotes neuronal excitability, while TNF-α participates in pain maintenance by damaging sensory nerve fibers, and continuous high expression of both may lead to chronic neuroinflammation (17). As an anti-inflammatory factor, the role of IL-10 in PHN is controversial (18). In this study, patients with PHN had significantly higher IL-10 levels than controls (18.18vs15.76 pg./mL, *p* = 0.004) and an 11% increase in PHN risk for every 1 pg./mL increase in levels (OR = 1.110). This might be related to the immunosuppressive effect of IL-10. Although IL-10 can reduce the inflammatory damage in the acute phase, its overexpression inhibits the antiviral immune response, resulting in the delayed clearance of the virus and interference with the nerve repair process (19). Animal experiments showed that after VZV infection, the viral load of IL-10 gene knockout mice was reduced, and the nerve damage was alleviated, indirectly supporting the promotion effect of IL-10 excess on PHN. Notably, IL-10 and Treg cells may have a synergistic effect ——Treg cells inhibit T cells by secreting IL-10, and the two together constitute an immunosuppressive microenvironment that hinders virus clearance and nerve repair. There is a complex interaction between inflammatory factors and t cell function (20). For example, IL-6 can promote Th17 cell differentiation, enhance the pro-inflammatory response, and inhibit Treg cell function. TNF-*α* can then induce CD8+ T cell apoptosis, further aggravating the immune response deficit (21). In this study, IL-6 and TNF-*α* together with CD4+/CD8+ ratio and Treg cell ratio entered the multi-factor model, suggesting that these indicators synergistically affected the occurrence of PHN through the “pro-inflammatory–immunosuppressive” network, that is, virus persistence and chronic inflammation were induced due to T cell dysfunction, while the imbalance of inflammatory factors in turn inhibited the immune response, forming a vicious circle.

The six independent risk factors (age, CD4+/CD8+ ratio, Treg cell ratio, IL-6, TNF-*α*, and IL-10) identified in this study covered the host age-related immune recession, imbalance of T cell subsets and imbalance of inflammatory factors, and formed a multi-level pathogenic network: age: as an immutable risk factor, aging accompanied by immune aging, manifested as decreased T cell proliferation and disordered secretion of inflammatory factors. Lower CD4+/CD8+ ratios, higher Treg cell ratios, and increased baseline levels of proinflammatory factors such as IL-6 and TNF-α in older patients, all of which lead to decreased viral clearance and impaired neural repair (22). CD4+/CD8+ ratio: a decrease in the ratio reflects either insufficient helper T cells or excessive activation of cytotoxic T cells, both of which may lead to an imbalanced antiviral response. Clinical monitoring of this ratio allows early recognition of immunocompromised patients and prompt intervention to enhance antiviral immunity. Proportion of Treg cells: Moderate Treg cells can reduce inflammatory damage in the acute phase, but overactivation inhibits immune clearance. How to find a balance between “controlling inflammation” and “maintaining antiviral immunity” may be a key target for PHN prevention. IL-6 and TNF-*α*: as the core mediators of neuroinflammation, the persistently high expression of both suggests the sustainability of nerve injury. Biological agents targeting IL-6 or TNF-α may become a new direction for the treatment of PHN, but further verification of their efficacy in acute phase interventions is needed (23). IL-10: its PHN-promoting effect suggests that anti-inflammatory treatment should avoid excessive inhibition of immune response. Monitoring IL-10 levels can help determine the state of immune repair and avoid delaying virus clearance due to excessive anti-inflammation.

The nomogram model integrates these indicators into a visual tool. The nomogram demonstrated robust discrimination (training AUC = 0.802, validation AUC = 0.790; Figure 3) and calibration (mean absolute error < 0.17; Figure 2), confirming its clinical utility for PHN risk stratification. This provides clinicians with an individualized evaluation tool, which is especially suitable for risk stratification of elderly patients and immunocompromised people.

There are some limitations in this study. First, the study samples were from a single center. While our nomogram integrates novel immunological predictors (Figure 1), its generalizability requires validation in multi-center cohorts (Table 1). The main reasons for not performing the external validation were that the patients included in the study cycle were from the same hospital, and the sample collection time span was from June 2022 to November 2024, and sufficient multi-center data had not been accumulated. In addition, potential influencing factors such as pain threshold and genetic factors (such as HLA genotype) were not included in the study, which may lead to slight bias on the prediction efficiency of the model. For index detection, only a single serum level at the time of admission was measured, and the changes of immune indexes during the course of disease were not dynamically tracked. In future studies, longitudinal data can be added to reveal the time-dependent pathogenic mechanism. Nevertheless, for the first time, in this study, T cell function and inflammatory factors are integrated to construct a PHN prediction model, which provides a new perspective for elucidating the immunologic mechanism of PHN. Future studies should explore: (1) dynamic T cell-cytokine interactions during PHN progression, (2) model-guided interventions (e.g., IL-6/TNF-*α* blockade), and (3) integration of genetic data to refine risk prediction. Through interdisciplinary integration, it is expected to open a new path for early intervention and individualized treatment of PHN.

In summary, in this study, we constructed an efficient prediction model of PHN by integrating T cell functional indicators with inflammatory factors, confirming the core role of immune imbalance in the occurrence of PHN. Despite its limitations, the results of this study provide an important basis for understanding the pathogenesis and clinical application of PHN, and further model verification and optimization in larger sample and multi-center studies are needed in the future to promote the development of accurate prevention strategies.

## Data Availability

The original contributions presented in the study are included in the article/supplementary material, further inquiries can be directed to the corresponding author.
